# Rheumatoid Arthritis and Risk of Depression in South Korea

**DOI:** 10.1001/jamanetworkopen.2024.1139

**Published:** 2024-03-05

**Authors:** Keun Hye Jeon, Kyungdo Han, Jinhyoung Jung, Chun Il Park, Yeonghee Eun, Dong Wook Shin, Hyungjin Kim

**Affiliations:** 1 Department of Family Medicine, CHA Gumi Medical Center, CHA University School of Medicine, Gumi, Republic of Korea; 2 Department of Statistics and Actuarial Science, Soongsil University, Seoul, Republic of Korea; 3 Department of Medical Statistics, College of Medicine, Catholic University of Korea, Seoul, Republic of Korea; 4 Department of Psychiatry, CHA Bundang Medical Center, CHA University School of Medicine, Seongnam, Republic of Korea; 5 Division of Rheumatology, Department of Internal Medicine, Kangbuk Samsung Hospital, Sungkyunkwan University School of Medicine, Seoul, Republic of Korea; 6 Department of Family Medicine/Supportive Care Center, Samsung Medical Center, Sungkyunkwan University School of Medicine, Seoul, Republic of Korea; 7 Department of Clinical Research Design and Evaluation, Samsung Advanced Institute for Health Science and Technology, Sungkyunkwan University, Seoul, Republic of Korea; 8 Department of Medicine, Samsung Medical Center, Sungkyunkwan University School of Medicine, Seoul, Republic of Korea; 9 Department of Medical Humanities, Samsung Medical Center, Sungkyunkwan University School of Medicine, Seoul, Republic of Korea

## Abstract

**Importance:**

Depression is among the most common comorbidities in rheumatoid arthritis (RA). There is a lack of data regarding the association of RA seropositivity and biologic agents with depression risk among individuals with RA.

**Objective:**

To investigate the risk of depression following RA diagnosis among patients in South Korea.

**Design, Setting, and Participants:**

This retrospective cohort study included 38 487 patients with RA and a comparison group of 192 435 individuals matched 1:5 for age, sex, and index date. Data were from the Korean National Health Insurance Service database. Participants were enrolled from 2010 to 2017 and were followed up until 2019. Participants who had previously been diagnosed with depression or were diagnosed with depression within 1 year after the index date were excluded. Statistical analysis was performed in May 2023.

**Exposures:**

Seropositive RA (SPRA) was defined with the *International Statistical Classification of Diseases and Related Health Problems, Tenth Revision (ICD-10)* codes M05 and enrollment in the Korean Rare and Intractable Diseases program. Seronegative RA (SNRA) was defined with *ICD-10* codes M06 (excluding M06.1 and M06.4) and a prescription of any disease-modifying antirheumatic drugs (DMARDs) for 270 days or more.

**Main Outcomes and Measures:**

Newly diagnosed depression (*ICD-10* codes F32 or F33).

**Results:**

The mean (SD) age of the total study population was 54.6 (12.1) years, and 163 926 individuals (71.0%) were female. During a median (IQR) follow-up of 4.1 (2.4-6.2) years, 27 063 participants (20 641 controls and 6422 with RA) developed depression. Participants with RA had a 1.66-fold higher risk of depression compared with controls (adjusted hazard ratio [aHR], 1.66 [95% CI, 1.61-1.71]). The SPRA group (aHR, 1.64 [95% CI, 1.58-1.69]) and the SNRA group (aHR, 1.73 [95% CI, 1.65-1.81]) were associated with an increased risk of depression compared with controls. Patients with RA who used biologic or targeted synthetic DMARDs (aHR, 1.33 [95% CI, 1.20-1.47]) had a lower risk of depression compared with patients with RA who did not use these medications (aHR, 1.69 [95% CI, 1.64-1.74]).

**Conclusions and Relevance:**

This nationwide cohort study found that both SPRA and SNRA were associated with a significantly higher risk of depression. These results suggest the importance of early screening and intervention for mental health in patients with RA.

## Key Points

**Question:**

Is there a higher risk of depression among specific populations of patients with rheumatoid arthritis (RA)?

**Findings:**

In this cohort study of 38 487 patients with RA and 192 435 individuals without RA, patients with seropositive RA and patients with seronegative RA were at a higher risk of depression compared with the control group, irrespective of socioeconomic, cardiometabolic, and behavioral factors. Patients with RA who received biological or targeted synthetic disease-modifying antirheumatic drugs (DMARDs) had a reduced risk of depression compared with patients who did not receive biological or targeted DMARDs.

**Meaning:**

These results suggest that clinicians should consistently screen all patients with RA for depression and provide comprehensive mental and physical health care.

## Teaser

This cohort study investigates the risk of depression following a rheumatoid arthritis diagnosis among patients in South Korea.

## Introduction

Rheumatoid arthritis (RA) is a prevalent autoimmune disease characterized by systemic inflammation.^1^ The chronic nature of the disease makes the disease a constant presence in the life of the patient, necessitating lifelong treatment and often leading to the development of many comorbidities. Depression is one of the most common comorbidities in RA.^2^ The prevalence of depression in individuals with RA is substantially higher compared with the general population, although estimates vary widely, ranging from 14% to 48%.^3^ The effect of depression on RA extends far beyond the burden of mental illness itself. The presence of comorbid depression in patients with RA has been associated with exacerbation of pain,^4^ increased disease activity,^5^ poor health-related quality of life,^5^ less frequent remission,^6,7^ increased risk of incident myocardial infarction,^8^ higher mortality rates,^9,10^ and greater utilization of health care services.^11^ Consequently, preventing and managing depression can be an effective approach to enhancing overall health and quality of life in these patients.

Several cohort studies reported on the risk of depression among patients with RA (eTable 1 in Supplement 1). A Korean study that examined the bidirectional association between RA and depression using a national sample cohort reported that RA increased the risk of depression but was limited by a relatively small sample size.^12^ Population-based cohort studies using the Taiwan National Health Insurance Research Database have found that the incidence of depression is 1.69 to 2.06 times higher in patients with RA compared with a control group,^13,14,15^ although these studies had not sufficiently adjusted for potential confounding factors. Previous studies have notable limitations: one study exclusively focused on individuals aged 60 years or older,^16^ and inconsistent results related to small sample sizes emerged from stratified analyses by age in other studies.^12,13^ Additionally, most previous studies relied solely on disease codes to identify RA cases, possibly introducing bias.^13,14,15,16,17^ Notably, cardiometabolic and behavioral factors, such as obesity, physical activity, smoking, and alcohol consumption, may influence the association between RA and depression, but these factors were not taken into consideration in previous studies.^12,13,14,15,16,17^ Furthermore, although the presence of rheumatoid factor (RF) or anticyclic citrullinated peptide antibodies (ACPA) can serve as markers of disease severity and provide insights into the risk of comorbidities, their effect on the development of depression in individuals with RA has remained unexplored. Moreover, there is a lack of data on the effect of biologic agents (eg, biologic disease-modifying antirheumatic drugs [DMARDs] and targeted synthetic DMARDs) on RA-associated depression.

In the present study, we investigated the association between RA and subsequent depression risk using a nationwide population-based cohort in South Korea, adjusting for cardiometabolic and behavioral factors that, to our knowledge, were not considered in previous studies. We conducted a rigorous diagnosis of RA, applying the diagnostic criteria via a separate registry for rare diseases in South Korea and the prescription of medication for RA. We also performed a series of subgroup analyses, including RA seropositivity and the type of DMARDs used, to define specific populations with higher risks of depression among the patients with RA.

## Methods

### Data Source and Study Setting

The South Korean National Health Insurance Service (NHIS) is a government-administered single insurer. The NHIS retains qualification data regarding demographics, health care use, diagnosis codes from the *International Statistical Classification of Diseases and Related Health Problems, Tenth Revision (ICD-10)*, medical treatment information, and a registry of cancer and rare and intractable disease (RID). The RID program, a special copayment reduction program for cancer and some other intractable diseases, relieves the large financial burden of patients with serious and rare diseases. The NHIS also offers a national health screening program every 2 years for insured individuals aged 40 years and above, as well as for all employees, regardless of age. This health screening program consists of a standard questionnaire on health behavior, anthropometric measurements, and laboratory tests.^18^ All these resources retained by the NHIS have been used to establish cohort data for various epidemiologic studies.^19,20^

This cohort study was approved by the institutional review board of Samsung Medical Center. The requirement for written informed consent was waived because of the anonymized feature of the data set. This study was designed and conducted according to Strengthening the Reporting of Observational Studies in Epidemiology (STROBE) reporting guideline.

### Study Population

The study included individuals who were first diagnosed with RA between 2010 and 2017. Seropositive RA (SPRA) was defined with *ICD-10* codes M05 and enrollment in the RID program. Registration in the RID program for SPRA requires a positive result for RF or ACPA and an official report from a physician documenting that the patient fulfills the classification criteria of RA; this approach is considered more valid than using *ICD-10* codes alone. However, only SPRA is registered in this registry, not seronegative RA (SNRA). SNRA was defined with *ICD-10* codes M06 (excluding M06.1 and M06.4) and a prescription of any DMARDs, including conventional synthetic DMARDs (csDMARDs), biologic DMARDs (bDMARDs), and targeted synthetic DMARDs (tsDMARDs) (eTable 2 in Supplement 1), for 270 days or more. The index date was defined as the date of registration in the RID program for SPRA and the administration of the first RA *ICD-10* code for SNRA.

**Figure 1.**
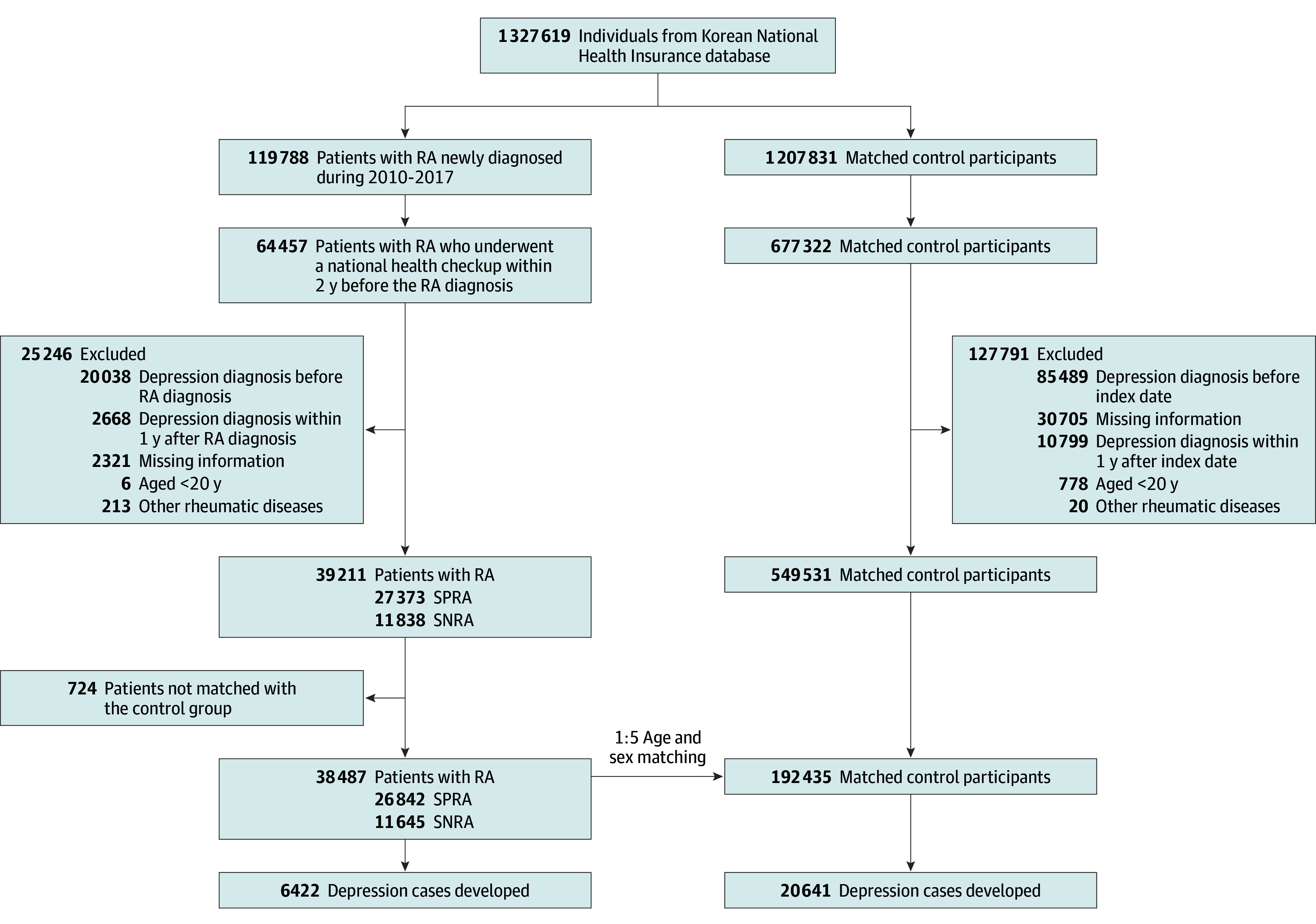
Flowchart of the Study Population RA indicates rheumatoid arthritis; SNRA, seronegative rheumatoid arthritis; SPRA, seropositive rheumatoid arthritis.

Out of the 119 789 identified cases of RA, we selected 64 457 patients who had undergone a national health screening within 2 years prior to their RA diagnosis. Among 64 457 participants, those who were younger than 20 years (n = 6), individuals who registered in the RID program for other rheumatic diseases (n = 213), and participants who had previously been diagnosed with depression (n = 20 038) were excluded. In addition, participants who were diagnosed with depression (n = 2668) within 1 year after the index date (called 1-year lag period) were excluded to minimize possible reverse causality. Finally, those whose records were missing any information (n = 2321) and who were not matched by age and sex with the control group (n = 724) were excluded. After these exclusions, a total of 38 487 patients with RA (26 842 with SPRA and 11 645 with SNRA) were included in the study. For the control group, 192 435 individuals without RA were matched (1:5) to RA cases based on age, sex, and index date (Figure 1). Race and ethnicity were not assessed because the Korean National Health Insurance Service database does not contain information about race or ethnicity. Information on covariates is described in eMethods in Supplement 1.

### Study Outcomes and Follow-Up

The end point of the study was newly diagnosed depression (F32 or F33), as used in previous studies.^20,21^ The study participants were followed from 1 year after RA diagnosis or corresponding index date to the date of depression diagnosis, death, or the end of the follow-up period (until December 31, 2019), whichever came first.

### Statistical Analysis

The baseline characteristics of the study participants were compared based on the presence of RA and the serologic status of RA. Continuous variables were presented as mean (SD) and categorical variables were presented as number and percentage. The incidence rates of depression were presented per 1000 person-years. The cumulative incidence of depression, based on RA status, serologic status of RA, and the type of DMARDs used, was estimated using the Kaplan-Meier method. Log-rank tests were applied to evaluate differences among the groups. We conducted Cox regression analyses to calculate adjusted hazard ratios (aHR) and 95% CIs for the risk of depression. A multivariable-adjusted proportional hazard model was applied: (1) model 1 was nonadjusted; (2) model 2 was adjusted for age, sex, smoking, alcohol drinking, physical activity, and income; (3) model 3 was further adjusted for body mass index, diabetes, hypertension, hyperlipidemia, and chronic kidney disease. The association between the type of DMARDs used and the risk of depression was also explored. Stratified analyses were performed by age, sex, health behaviors, and comorbidities. To compare the risk of depression according to age and sex, we analyzed differences in restricted mean survival time (RMST) between groups. The RMST is the mean length of time (days) that patients remain free of an event until a certain point in time. Statistical analyses were performed in May 2023 using SAS version 9.4 (SAS Institute Inc), and 2-sided *P* < .05 was considered statistically significant.

## Results

### Baseline Characteristics

Table 1 represents the baseline characteristics of the study participants according to the presence of RA and the serologic status of RA. Among a total of 230 922 study participants at the index date, 163 926 individuals (71.0%) were female, and the mean (SD) age was 54.6 (12.1) years. When compared with those without RA, the patients with RA tended to be nondrinkers, be less engaged in regular exercises, and be less likely to have obesity. Patients in the SPRA group were more likely to be older (55.6 vs 52.3 years), female, more likely to be nondrinkers, and less likely to have obesity than those in the SNRA group.

**Table 1.** Baseline Characteristics of Study Participants

Characteristic	Total	RA status			Serologic status of RA		
		No	Yes	*P* value	SNRA	SPRA	*P* value
Participants, No.	230 922	192 435	38 487	NA	11 645	26 842	NA
Age, mean (SD), y	54.6 (12.1)	54.6 (12.1)	54.6 (12.1)	NA	52.3 (12.8)	55.6 (11.6)	NA
20-39	23 550 (10.2)	19 625 (10.2)	3925 (10.2)	>.99	1905 (16.4)	2020 (7.5)	<.001
40-59	127 242 (55.1)	106 035 (55.1)	21 207 (55.1)		6336 (54.4)	14 871 (55.4)	
≥60	80 130 (34.7)	66 775 (34.7)	13 355 (34.7)		3404 (29.2)	9951 (37.1)	
Sex							
Male	66 996 (29.0)	55 830 (29.0)	11 166 (29.0)	>.99	3928 (33.7)	7238 (27.0)	<.001
Female	163 926 (71.0)	136 605 (71.0)	27 321 (71.0)		7717 (66.3)	19 604 (73.0)	
Income							
Low ≥25% Medicaid group	51 583 (22.3)	43 205 (22.5)	8378 (21.8)	.003	2306 (19.8)	6072 (22.6)	<.001
Smoking status							
Never or former smoking	201 349 (87.2)	167 750 (87.2)	33 599 (87.2)	.50	NA	NA	.97
Current smoking	29 573 (12.8)	24, 685 (12.8)	4888 (12.7)		1, 480 (12.7)	3408 (12.7)	
Alcohol consumption							
None	152 973 (66.2)	125 642 (65.3)	27 331 (71.0)	<.001	NA	NA	<.001
>0 g/d	77 949 (33.8)	66 793 (34.7)	11 156 (29.0)		3835 (32.9)	7321 (27.3)	
Physical activity							
None	185 542 (80.3)	153 906 (80.0)	31 636 (82.2)	<.001	NA	NA	.008
Regular	45 380 (19.7)	38 529 (20.0)	6851 (17.8)		2165 (18.6)	4686 (17.5)	
BMI							
<25	158 212 (68.5)	130 665 (67.9)	27 547 (71.6)	<.001	NA	NA	<.001
≥25	72 710 (31.5)	61 770 (32.1)	10 940 (28.4)		3582 (30.8)	7358 (27.4)	
Diabetes	25 438 (11.02)	21 356 (11.1)	4082 (10.6)	.005	1233 (10.6)	2849 (10.6)	.94
Hypertension	75 246 (32.6)	61 785 (32.1)	13 461 (35.0)	<.001	4206 (36.1)	9255 (34.5)	.002
Dyslipidemia	63 066 (27.3)	52 660 (27.4)	10 406 (27.0)	.19	3324 (28.5)	7082 (26.4)	<.001
Chronic kidney disease	12 971 (5.6)	10 270 (5.3)	2701 (7.0)	<.001	895 (7.7)	1806 (6.7)	.001

Abbreviations: BMI, body mass index (calculated as weight in kilograms divided by height in meters squared); NA, not applicable; SNRA, seronegative rheumatoid arthritis; SPRA, seropositive rheumatoid arthritis.

### Risk of Depression According to the Presence of RA and RA Serologic Status

**Figure 2.**
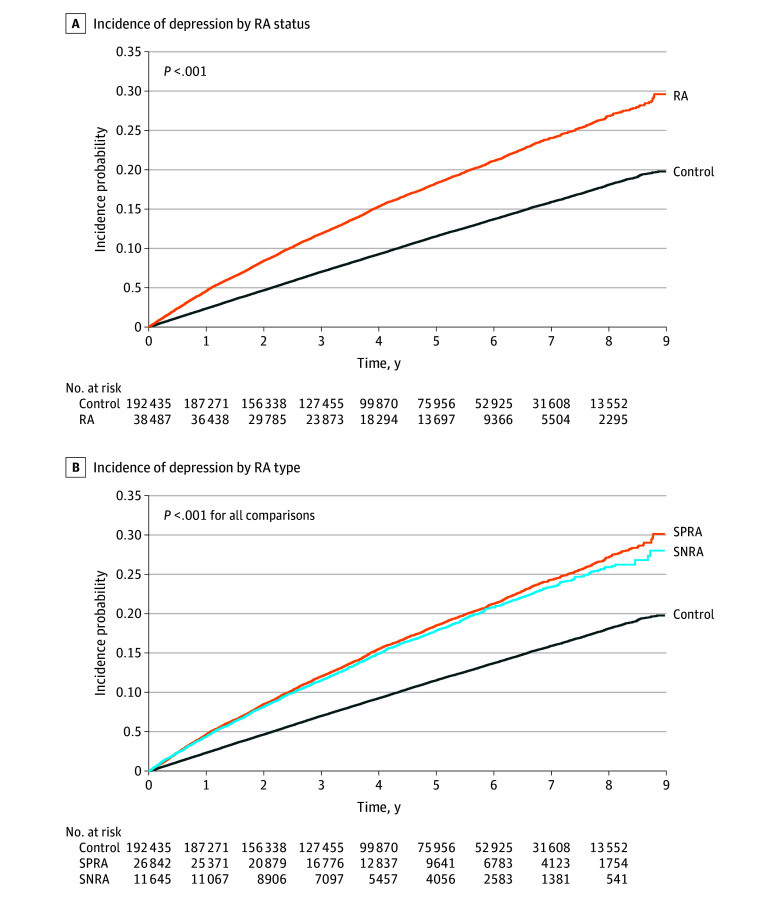
Cumulative Incidence of Depression According to Rheumatoid Arthritis (RA) Status and Serologic Status of RA RA indicates rheumatoid arthritis; SNRA, seronegative rheumatoid arthritis; SPRA, seropositive rheumatoid arthritis.

During a median (IQR) follow-up of 4.1 (2.4-6.2) years after a 1-year lag period, 27 063 participants (6422 in the RA group and 20 641 in the control group) were newly diagnosed with depression (Figure 2). Compared with the control group, patients with RA showed a higher risk for depression (aHR, 1.66 [95% CI, 1.61-1.71]) (Table 2). Both the SPRA group (aHR, 1.64 [95% CI, 1.58-1.69]) and SNRA group (aHR, 1.73 [95% CI, 1.65-1.81]) were associated with an increased risk of depression compared with the control. When the SNRA group was used as the reference group, the aHR for depression was 0.96 (95% CI, 0.91-1.01) in the SPRA group.

**Table 2.** Risk of Depression According to RA Status and Serologic Status of RA^a^

	No.	Events, No.	Duration, PY	Incidence rate (per 1000 PY)	HR (95% CI)		
					Model 1	Model 2	Model 3
By RA status							
Control	192 435	20 641	841 495.3	24.53	1 [Reference]	1 [Reference]	1 [Reference]
RA	38 487	6422	158 423.7	40.54	1.65 (1.61-1.70)	1.67 (1.62-1.71)	1.66 (1.61-1.71)
By RA status and seropositivity							
Control	192 435	20 641	841 495.3	24.53	1 [Reference]	1 [Reference]	1 [Reference]
SNRA	11 645	1846	46 776.5	39.46	1.61 (1.53-1.69)	1.74 (1.66-1.83)	1.73 (1.65-1.81)
SPRA	26 842	4576	111 647.2	40.99	1.67 (1.62-1.73)	1.64 (1.58-1.69)	1.64 (1.58-1.69)
By seropositivity							
SNRA	11 645	1846	46 776.5	39.46	1 [Reference]	1 [Reference]	1 [Reference]
SPRA	26 842	4576	111 647.2	40.99	1.04 (0.99-1.10)	0.95 (0.90-1.00)	0.96 (0.91-1.01)

Abbreviations: PY, person-years; RA, rheumatoid arthritis; SNRA, seronegative RA; SPRA, seropositive RA.

^a^ Model 1 was nonadjusted; model 2 was adjusted for age, sex, smoking, alcohol drinking, physical activity, and income; model 3 was adjusted additionally for body mass index, diabetes, hypertension, hyperlipidemia, and chronic kidney disease.

### Type of DMARDs Used and Risk of Depression

Among the 6422 patients diagnosed with depression in the RA group, 402 patients were prescribed bDMARDs or tsDMARDs, and 6020 patients were prescribed csDMARDs only, with no exposure to bDMARDs or tsDMARDs (eTable 3 in Supplement 1). The cumulative incidence of depression was consistently lower in the group of patients with RA who received bDMARDs or tsDMARDs than those who did not over the entire follow-up period (eFigure in Supplement 1). Patients with RA who received bDMARDs or tsDMARDs (aHR, 1.33 [95% CI, 1.20-1.47) had a lower risk of depression compared with patients with RA who did not receive bDMARDs or tsDMARDs (aHR, 1.69 [95% CI, 1.64-1.74]).

### Stratified Analysis

Stratified analyses by age, sex, income, body mass index, health behaviors, and comorbidities showed consistent results with the main findings (eTables 4 and 5 in Supplement 1). The RMST differences varied across age groups, with a greater difference observed in the group aged 60 years and older due to the higher incidence rate of depression (eTable 4 in Supplement 1).

## Discussion

In this nationwide cohort study, we observed that individuals with RA had a 1.66-fold higher risk of depression compared with those without RA. Furthermore, patients with both the SPRA and the SNRA exhibited an increased risk of depression, whereas there was no statistically significant difference in the risk of depression based on RA serologic status.

Our findings revealed a higher risk of depression in the RA group compared with the matched control participants, which is consistent with previous studies^12,13,14,15,16,17^ reporting a 1.20-fold to 2.06-fold increased risk of depression among patients with RA compared with the control group. The current study demonstrates several strengths in its methods compared with previous research. First, while most previous studies relied solely on disease codes to identify RA cases, our study used a combination of disease codes, RID program enrollment, and prescription data for DMARDs to define RA, thereby minimizing the potential for misclassification. Second, previous studies did not take into account important confounding variables such as body mass index, smoking, alcohol consumption, and physical activity. In contrast, we adjusted sequentially for socioeconomic, cardiometabolic, and behavioral factors, as well as comorbidities in the multivariate-adjusted model, and we found that the results were consistent across all models. Third, in our study, to minimize possible reverse causality, the participants were followed from 1 year after the index date. To our knowledge, with 38 487 RA patients, the present study is the largest to date examining the association of RA with depression and providing solid evidence supporting the positive correlation between RA and the risk of depression.

To the best of our knowledge, the current study is the first investigation to find an association between RA and subsequent depression risk based on RA seropositivity. The findings of the study indicate that both SPRA and SNRA patients are at a higher risk of depression compared with the control group. Although SNRA is generally considered a milder form of the disease than SPRA, it exhibits varying clinical outcomes.^22,23^ In fact, patients with SNRA require a greater number of clinical symptoms to meet the American College of Rheumatology/European League Against Rheumatism criteria for RA classification compared with patients with SPRA.^24^ Previous studies indicate that patients with SNRA are less satisfied with their treatment,^25^ more likely to complain of persistent pain,^26^ and have a higher likelihood of developing concomitant fibromyalgia.^27^ A meta-analysis has reported that the association between RA and depression was proportionally related to the level of pain experienced by patients with RA.^28^ Various factors, including repeated physical pain, fatigue, gradual loss of function, and disease-related emotional and quality-of-life problems, could contribute to the risk of depression in patients with RA regardless of RA serologic status.

The specific biological mechanisms that underlie the association between RA and depression have not yet been fully understood. However, long-term systemic inflammation has been suggested as a plausible explanation, supported by the association of proinflammatory cytokines such as tumor necrosis factor (TNF)-α, interleukin (IL)-1β, IL-6, and other chemokines with both RA and depression.^29^ These cytokines cross the blood-brain barrier, interacting with the brain and triggering microglial activation, releasing proinflammatory factors.^30,31,32^ Neural pathways also transmit peripheral inflammation signals to the central nervous system (CNS), inducing CNS inflammation. This process leads to hypothalamic-pituitary-adrenal axis overactivation,^33^ changes in brain structure and function, elevated glutamate levels, reduced γ-aminobutyric acid expression, and altered brain-derived neurotrophic factors, collectively contributing to the development of depression.^34^ Based on the Kaplan-Meier curves from our study, it can be inferred that depression occurs similarly in the SPRA and SNRA groups initially. This similarity is likely due to the psychological burden of receiving an RA diagnosis and experiencing uncontrolled pain in the early stages of RA. However, as time progresses, the effect of inflammation seems to contribute to a marginally higher incidence of depression in patients with SPRA compared with patients with SNRA. To fully understand the underlying mechanisms behind these findings, further research with a longer follow-up period than our current study is warranted.

We found that patients with RA who used bDMARDs or tsDMARDs had a reduced risk of depression compared with those who did not use these medications. Theoretically, these agents have the potential to improve comorbidities linked to RA through better control of systemic inflammation. However, there is a lack of data on the effect of biologic agents on depression. A study conducted in Japan found that combined infliximab and methotrexate treatment was more effective in improving the depressive state of patients with RA compared with methotrexate alone.^35^ In a Taiwan cross-sectional study, authors found a significantly lower risk of depression among patients receiving etanercept compared with the nonbiologic group.^36^ Furthermore, the risk for depression was significantly reduced among patients with RA who responded to TNF-inhibitor therapy compared with those who did not respond to such therapy.^37^ Some studies have suggested that the administration of tocilizumab, an anti-IL-6 receptor monoclonal antibody, may improve depression in individuals with RA.^38,39^ However, in a recent review, Matcham et al^40^ argued that relying solely on biologic agents such as bDMARDs and tsDMARDs is unlikely to yield significant improvements in mental health outcomes for patients with RA. The efficacy of biological agents for the treatment of RA-associated depression is still controversial and further studies are needed to explore the potential benefit.

There was no significant interaction between RA and demographic characteristics on the risk of depression. While it has been reported that early-onset (onset <60 years of age) and elderly-onset RA (onset ≥60 years of age) exhibit differences in the course and prognosis of the disease,^41^ in our age-stratified analyses, the association between RA and the risk of depression was similar among all age groups. Regardless of the age of onset, RA appears to affect the risk of depression. Additionally, we found no significant difference in the risk of depression between male and female patients with RA. A recent systematic review and meta-analysis of 3 longitudinal studies also supported these findings, showing that the subgroup analysis by sex revealed similar results for female and male individuals.^42^ Given the female predominance in RA, male patients remain largely underrepresented and understudied, with comparatively smaller sample sizes. Our study included a substantial number of male RA patients (n = 11 166) and provides strong evidence to support our findings.

### Limitations

There were limitations to our study. First, RA disease activity was not accessible, resulting in a limited evaluation of the severity of RA. Second, although our models were adjusted for various potential confounders, unmeasured factors, such as social support factors and family history, might still distort the results. Third, information regarding the level of depression among study participants at the index dates is unavailable. There is a possibility that individuals with RA might undergo undiagnosed or subclinical depression due to prodromal symptoms, pains, limitations on social activities, or heightened psychosocial stressors, even before receiving a clinical diagnosis for RA. Fourth, our study participants were limited to health screening participants and may have been healthier and more engaged in having a healthy lifestyle than the general population.

## Conclusions

This nationwide cohort study found a strong association between RA and an increased risk of depression, regardless of age, sex, behavioral factors, and RA serologic status. Therefore, clinicians should consistently screen all RA patients for depression and provide comprehensive health care that addresses both mental and physical well-being.

## Notes

Supplement 1. **eMethods.** Information on Covariates in the Study **eTable 1.** Previous Studies on the Relationship Between Rheumatoid Arthritis (RA) and the Incidence of Depression **eTable 2.** Disease-Modifying Anti-Rheumatic Drugs Used in the Definition of Diseases **eTable 3.** The Risk of Depression According to Exposure to Biologic DMARDs (bDMARDs) and Targeted Synthetic DMARDs (tsDMARDs) in Patients With Rheumatoid Arthritis (RA) **eTable 4.** Stratified Analyses of the Association Between RA Status and Depression Risk by Age and Sex **eTable 5.** Stratified Analyses of the Association Between RA Status and Depression Risk by Income, Health Behaviors, and Comorbidities **eFigure.** (A) Unadjusted and (B) Adjusted Kaplan–Meier Curve for the Incidence Depression According to the Type of Disease-Modifying Anti-Rheumatic Drugs (DMARDs) Used

Supplement 2.
Data Sharing Statement
