# Supplementary material for: Rheumatoid Arthritis and Risk of Depression in South Korea
Source: JAMA Netw Open. 2024 Mar 5;7(3):e241139. doi: 10.1001/jamanetworkopen.2024.1139 (PMC10915683; doi:10.1001/jamanetworkopen.2024.1139)
Supplement: Supplement 1. — eMethods. Information on Covariates in the Study eTable 1. Previous Studies on the Relationship Between Rheumatoid Arthritis (RA) and the Incidence of Depression eTable 2. Disease-Modifying Anti-Rheumatic Drugs Used in the Definition of Diseases eTable 3. The Risk of Depression According to Exposure to Biologic DMARDs (bDMARDs) and Targeted Synthetic DMARDs (tsDMARDs) in Patients With Rheumatoid Arthritis (RA) eTable 4. Stratified Analyses of the Association Between RA Status and Depression Risk by Age and Sex eTable 5. Stratified Analyses of the Association Between RA Status and Depression Risk by Income, Health Behaviors, and Comorbidities eFigure. (A) Unadjusted and (B) Adjusted Kaplan–Meier Curve for the Incidence Depression According to the Type of Disease-Modifying Anti-Rheumatic Drugs (DMARDs) Used [file jamanetwopen-e241139-s001.pdf]

Supplemental Online Content

Jeon KH, Han K, Jung J, et al. Rheumatoid arthritis and risk of depression in South Korea. *JAMA Netw Open.* 2024;7(3):e241139. doi:10.1001/jamanetworkopen.2024.1139

eMethods. Information on Covariates in the Study

eTable 1. Previous Studies on the Relationship Between Rheumatoid Arthritis (RA) and the Incidence of Depression

eTable 2. Disease-Modifying Anti-Rheumatic Drugs Used in the Definition of Diseases

eTable 3. The Risk of Depression According to Exposure to Biologic DMARDs (bDMARDs) and Targeted Synthetic DMARDs (tsDMARDs) in Patients With Rheumatoid Arthritis (RA)

eTable 4. Stratified Analyses of the Association Between RA Status and Depression Risk by Age and Sex

eTable 5. Stratified Analyses of the Association Between RA Status and Depression Risk by Income, Health Behaviors, and Comorbidities

eFigure 1. (A) Unadjusted and (B) Adjusted Kaplan–Meier Curve for the Incidence Depression According to the Type of Disease-Modifying Anti-Rheumatic Drugs (DMARDs) Used

This supplemental material has been provided by the authors to give readers additional information about their work.

© 2024 Jeon KH et al. *JAMA Network Open.*

eMethods. Information on Covariates in the study

Income level was categorized into quartiles, in which those covered by Medical Aid were merged into the lowest-income quartile group. Information about health-related behavior was obtained using a self-administered questionnaire. Smoking status was categorized as never/ex-smoker or current smoker, and alcohol consumption was classified as non-drinker or drinker (>0 g/day). Regular exercise was defined as performing > 30 minutes of moderate physical activity at least five times per week or > 20 minutes of strenuous physical activity at least three times per week, as used in previous studies.[1-3] Body mass index (BMI) was calculated using the body weight (kg) divided by height in meters squared (m2). Comorbidities were defined based on medical claims according to the ICD-10 codes (for hypertension, I10-I13 or I15; for diabetes, E11-E14; for dyslipidemia, E78; and for chronic kidney disease, N18 or N19).

1. Park MJ, Yoo J, Han K, Shin DW, Fava M, Mischoulon D, Jeon HJ. High body weight variability is associated with increased risk of depression: a nationwide cohort study in South Korea. Psychol Med. 2023 Jun;53(8):3719-3727.
2. Yook V, Yoo J, Han K, Fava M, Mischoulon D, Park MJ, Kim H, Jeon HJ. Association between pre-pregnancy tobacco smoking and postpartum depression: A nationwide cohort study. J Affect Disord. 2022 Nov 1;316:56-62.
3. Kang J, Eun Y, Jang W, Cho MH, Han K, Jung J, Kim Y, Kim GT, Shin DW, Kim H. Rheumatoid Arthritis and Risk of Parkinson Disease in Korea. JAMA Neurol. 2023 Jun 1;80(6):634-641.

© 2024 Jeon KH et al. *JAMA Network Open.*

eTable 1. Previous studies on the relationship between Rheumatoid arthritis (RA) and the incidence of depression

**Study, country**	**Index period**	**No. of participants**	**Matching**	**Range of age**	**Mean or median age (years)**	**Female (%)**	**Mean or median follow-up (years)**	**RA Assessment**	**Depression Assessment**	**No. of Outcomes (Incidence rate)**	**Adjustment**	**Adjusted HR (95% CI)**	
Kim et al (2020), South Korea	2002-2013	RA<br>7,385<br>Control<br>29,540	age, sex, income, region of residence	≥20	not reported	76.8	RA<br>7.25±3.60<br>Control<br>7.29±3.63	ICD 10 code: M05, M06 and a prescription for a biologic agent or any DMARD	ICD-10: F31-33, treated 2 or more times	RA 408<br>(not reported)<br>Control 1,246<br>(not reported)	Charlson comorbidity index (CCI)	Overall	**1.20 (1.07-1.34)**
												Age<br><30<br>30–59<br>≥60	 0.81 (0.45–1.45)<br>**1.17 (1.01–1.36)**<br>**1.29 (1.08–1.55)**
												Sex<br>Male<br>Female	 1.26 (0.95–1.67)<br>**1.19 (1.05–1.35)**
Marrie et al (2018), Canada	1989-2012	RA<br>10,206<br>Control<br>50,960	age, sex, region of residence	≥18	RA<br>53.7±16	72.2	RA<br>9.19<br>Control<br>9.05	ICD-9-CM code: 714 /ICD 10 code: M05, M06	ICD-9-CM: 296.2, 296.3 298.0, 300.4 or 311 /ICD-10: F32-34	not reported	Number physician visits, follow up duration	Overall	**1.46 (1.35–1.58)**
Drosselmeyer et al (2017), Germany	2009-2013	RA<br>7,301<br>Control<br>7,301	age, sex, depression diagnosis in the past, follow-up duration	≥60	RA<br>72.2±7.60<br>Control<br>72.2±7.60	65.1	RA<br>3.40±1.20<br>Control<br>3.40±1.20	ICD 10 code: M05, M06	ICD-10: F32 or F33	RA 1,606<br>(not reported)<br>Control 1,044<br>(not reported)	Comorbidity[a]	Overall	**1.55 (1.38-1.74)**
Lu et al (2016), Taiwan	1998-2011	RA<br>8,331<br>Control<br>15,456	age, sex, index year	20-80	RA<br>54.6±14.0<br>Control<br>54.4±14.0	67.8	RA<br>7.33<br>Control<br>7.77	ICD-9-CM code: 714, 3 ambulatory diagnoses or 1 admission diagnosis	ICD-9-CM: 296.2, 296.3 300.4 or 311	RA 958<br>(15.69 per 1,000 PY)<br>Control 1,075<br>(8.95 per 1,000 PY)	Urbanization level, income, comorbidity[b]	Overall	**1.69 (1.51-1.89)**
												Sex & Age<br>Female<br>≤40<br>41-60<br>>60<br>Male<br>≤40<br>41-60<br>>60	 **1.73 (1.50–1.84)**<br>**2.14 (1.62–2.69)**<br>**1.60 (1.35–1.90)**<br>**1.58 (1.37–1.82)**<br>**1.65 (1.33–1.94)**<br>**2.76 (1.83–4.59)**<br>**1.59 (1.17–2.17)**<br>**1.33 (1.01–1.75)**
Lin et al (2015), Taiwan	2000-2004	RA<br>3,698<br>Control<br>7,396	age, sex, index year	≥18	RA<br>55.8±15.2<br>Control<br>55.8±15.2	65.8	RA<br>9.46<br>Control<br>9.66	ICD-9-CM code: 714, 2 ambulatory diagnoses or 1 admission diagnosis	ICD-9-CM: 296.2, 296.3 300.4 or 311	RA 413<br>(11.80 per 1,000 PY)<br>Control 492<br>(6.89 per 1,000 PY)	Urbanization level, income, medication usage, comorbidity[c]	Overall	**1.74 (1.48-1.95)**

© 2024 Jeon KH et al. *JAMA Network Open.*

Wang et al (2014), Taiwan	2000-2008	RA 3,657 Control 14,628	age, sex, index year	≥20	RA 51.0 Control 51.0	72.8	RA 4.72 Control 4.92	ICD-9-CM code: 714	Psychiatrist diagnosed depressive disorder	RA 205 (11.2 per 1,000 PY) Control 384 (5.1 per 1,000 PY)	Urbanization level, income, comorbidity[d]	Overall	**2.06 (1.73–2.44)**

RA, Rheumatoid arthritis; PY, person-years; HR, hazard ratio; CI, confidence interval

[a] hypertension, diabetes mellitus, myocardial infarction, coronary heart disease, heart failure, stroke, dementia, cancer, osteoporosis, inflammatory poly arthropathies excluding RA, and osteoarthritis

[b] hypertension, diabetes mellitus, chronic kidney disease, heart disease, stroke, cancer, alcohol dependence syndrome, and tobacco use

[c] hypertension, diabetes mellitus, chronic kidney disease, heart disease, stroke, and cancer

[d] hypertension, diabetes mellitus, dyslipidemia, coronary artery disease, congestive heart failure, cirrhosis, hyperthyroidism, hypothyroidism, cerebrovascular disease, and cancer

© 2024 Jeon KH et al. *JAMA Network Open.*

eTable 2. Disease-Modifying Anti-Rheumatic Drugs Used in the Definition of Diseases.

Category	Drugs
csDMARD	Methotrexate, Hydroxychloroquine, Leflunomide, Sulfasalazine, Tacrolimus, Cyclosporine, D-penicillamine, Bucillamine, Azathioprine, Minocycline, Mizoribine
bDMARD	Adalimumab, Etanercept, Infliximab, Golimumab, Rituximab, Abatacept, Tocilizumab
tsDMARD	Tofacitinib

csDMARD, conventional synthetic disease-modifying anti-rheumatic drug; bDMARD, biologic disease-modifying anti-rheumatic drug; tsDMARD, targeted synthetic disease-modifying anti-rheumatic drug

© 2024 Jeon KH et al. *JAMA Network Open.*

eTable 3. The risk of Depression According to Exposure to Biologic DMARDs (bDMARDs) and Targeted synthetic DMARDs (tsDMARDs) in Patients with Rheumatoid Arthritis (RA)

Subgroup	N	Event (N)	Duration (PY)	Incidence rate (per 1,000 PY)	aHR (95% CI)
By RA status and bDMARDs and tsDMARDs use					
Control	192,435	20,641	841,495.3	24.53	1 (Ref.)
RA, bDMARDs or tsDMARDs unexposed	35,439	6,020	144,646.8	41.62	1.69 (1.64-1.74)
RA, bDMARDs or tsDMARDs exposed	3,048	402	13,777.0	29.18	1.33 (1.20-1.47)
By RA seropositivity and bDMARDs and tsDMARDs use					
Control	192,435	20,641	841,495.3	24.53	1 (Ref.)
SNRA, bDMARDs or tsDMARDs unexposed	11,222	1,806	44,958.5	40.17	1.73 (1.65-1.82)
SNRA, bDMARDs or tsDMARDs exposed	423	40	1,818.0	22.00	1.48 (1.08-2.02)
SPRA, bDMARDs or tsDMARDs unexposed	24,217	4,214	99,688.3	42.27	1.67 (1.62-1.73)
SPRA, bDMARDs or tsDMARDs exposed	2,625	362	11,959.0	30.27	1.32 (1.19-1.46)

RA, Rheumatoid arthritis; PY, person-years; aHR, adjusted hazard ratio; CI, confidence interval; SNRA, seronegative RA; SPRA, seropositive RA

aHRs were adjusted for age, sex, smoking, alcohol drinking, physical activity, income, body mass index, diabetes mellitus, hypertension, hyperlipidemia, and chronic kidney disease.

© 2024 Jeon KH et al. *JAMA Network Open.*

eTable 4. Stratified analyses of the association Between RA Status and Depression Risk by Age and Sex

Subgroup	RA	N	Event (N)	Duration (PY)	Incidence rate (per 1,000 PY)	aHR (95% CI)	*P* for interaction	Difference in RMST [a] (95% CI), day	*P value*
Age									
20-39	Control	19,625	1,003	90,734.8	11.05	1 (Ref.)	0.81	Ref.	
	RA	3,925	333	17,609.3	18.91	1.70 (1.50-1.93)		-91.8 (-123.5, -60.2)	<.0001
40-59	Control	106,035	9,583	477,341.6	20.08	1 (Ref.)		Ref.	
	RA	21,207	3,074	91,073.5	33.75	1.67 (1.61-1.74)		-142.3 (-161.5, -123.2)	<.0001
≥60	Control	66,775	10,055	273,418.8	36.78	1 (Ref.)		Ref.	
	RA	13,355	3,015	49,740.9	60.61	1.65 (1.58-1.72)		-274.0 (-306.6, -241.5)	<.0001
Sex									
Male	Control	55,830	4,943	23,6410.4	20.91	1 (Ref.)	0.13	Ref.	
	RA	11,166	1,587	44,312.9	35.81	1.73 (1.63-1.83)		-192.5 (-219.2, -165.8)	<.0001
Female	Control	136,605	15,698	605,084.8	25.94	1 (Ref.)		Ref.	
	RA	27,321	4,835	114,110.9	42.37	1.64 (1.59-1.69)		-171.5 (-190.7, -152.4)	<.0001

RA, Rheumatoid arthritis; PY, person-years; aHR, adjusted hazard ratio; CI, confidence interval; RMST, restricted mean survival time

aHRs were adjusted for age, sex, smoking, alcohol drinking, physical activity, income, body mass index, diabetes mellitus, hypertension, hyperlipidemia, and chronic kidney disease.

[a] Truncated time was 8 years.

© 2024 Jeon KH et al. *JAMA Network Open.*

eTable 5. Stratified analyses of the association Between RA Status and Depression Risk by Income, Health Behaviors, and Comorbidities

Subgroup	RA	N	Event (N)	Duration (PY)	Incidence rate (per 1,000 PY)	aHR (95% CI)	*P* for interaction
Income							
Low 25% + Medicaid group	Control	149,230	15,780	653,380.4	24.15	1 (Ref.)	0.60
	RA	30,109	4,976	124,693.8	39.91	1.65 (1.60-1.71)	
Above 25%	Control	43,205	4,861	188,114.8	25.84	1 (Ref.)	
	RA	8,378	1,446	33,729.9	42.87	1.68 (1.59-1.79)	
Smoking							
Nonsmoker	Control	167,750	18,373	735,017.4	25.00	1 (Ref.)	0.28
	RA	33,599	5,675	138,742.5	40.90	1.65 (1.60-1.70)	
Current smoker	Control	24,685	2,268	106,477.9	21.30	1 (Ref.)	
	RA	4,888	747	19,681.2	37.96	1.73 (1.60-1.88)	
Alcohol consumption							
Nondrinker	Control	125,642	15,150	552,967.2	27.40	1 (Ref.)	<.001
	RA	27,331	4,881	113,043.4	43.18	1.61 (1.56-1.67)	
Drinker	Control	66,793	5,491	288,528.0	19.03	1 (Ref.)	
	RA	11,156	1,541	45,380.3	33.96	1.82 (1.72-1.93)	
Physical activity							
None	Control	153,906	16,652	675,793.1	24.64	1 (Ref.)	0.76
	RA	31,636	5,321	130,719.3	40.71	1.66 (1.61-1.72)	
Regular	Control	38,529	3,989	165,702.2	24.07	1 (Ref.)	
	RA	6,851	1,101	27,704.5	39.74	1.64 (1.54-1.76)	
Body mass index							
<25	Control	130,665	13,863	574,280.8	24.14	1 (Ref.)	0.09
	RA	27,547	4,507	114,399.8	39.40	1.63 (1.58-1.69)	
≥25	Control	61,770	6,778	267,214.5	25.37	1 (Ref.)	
	RA	10,940	1,915	44,023.9	43.50	1.72 (1.64-1.81)	
Diabetes							
No	Control	171,079	17,644	754,098.6	23.40	1 (Ref.)	0.72
	RA	34,405	5,557	143,376.7	38.76	1.66 (1.61-1.71)	
Yes	Control	21,356	2,997	87,396.6	34.29	1 (Ref.)	
	RA	4,082	865	15,047.1	57.49	1.68 (1.56-1.81)	
Hypertension							
No	Control	130,650	12,256	577,829.8	21.21	1 (Ref.)	0.08
	RA	25,026	3,619	105,523.0	34.30	1.62 (1.57-1.69)	
Yes	Control	61,785	8,385	263,665.4	31.80	1 (Ref.)	
	RA	13,461	2,803	52,900.7	52.99	1.71 (1.64-1.79)	
Dyslipidemia							
No	Control	139,775	14,019	625,783.2	22.40	1 (Ref.)	0.40

© 2024 Jeon KH et al. *JAMA Network Open.*

	RA	28,081	4,390	118,770.3	36.96	1.65 (1.59-1.70)	
Yes	Control	52,660	6,622	215,712.0	30.70	1 (Ref.)	
	RA	10,406	2,032	39,653.4	51.24	1.69 (1.61-1.78)	
Chronic kidney disease							
No	Control	182,165	18,943	795,715.6	23.81	1 (Ref.)	0.03
	RA	35,786	5,855	147,589.4	39.67	1.68 (1.63-1.73)	
Yes	Control	10,270	1,698	45,779.6	37.09	1 (Ref.)	
	RA	2,701	567	10,834.3	52.33	1.50 (1.36-1.65)	

RA, Rheumatoid arthritis; PY, person-years; aHR, adjusted hazard ratio; CI, confidence interval

aHRs were adjusted for age, sex, smoking, alcohol drinking, physical activity, income, body mass index, diabetes mellitus, hypertension, hyperlipidemia, and chronic kidney disease.

© 2024 Jeon KH et al. *JAMA Network Open.*

eFigure 1. (A) Unadjusted and (B) adjusted Kaplan–Meier curve for the incidence depression according to the type of Disease-Modifying Anti-Rheumatic Drugs (DMARDs) used

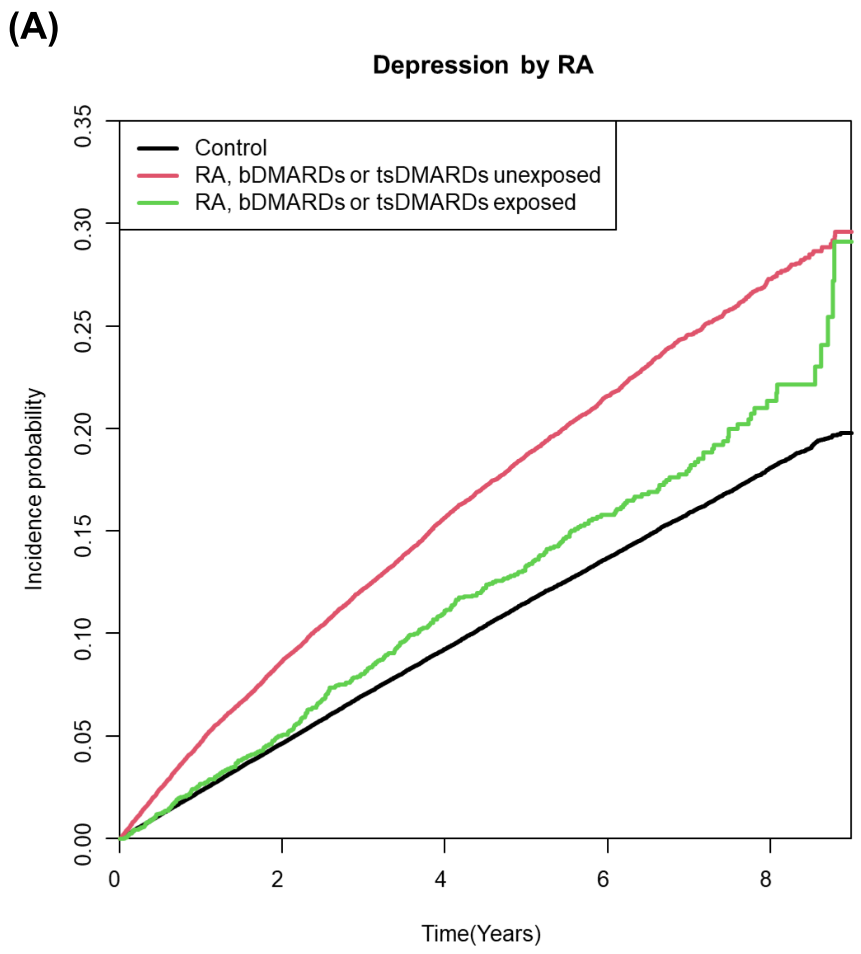

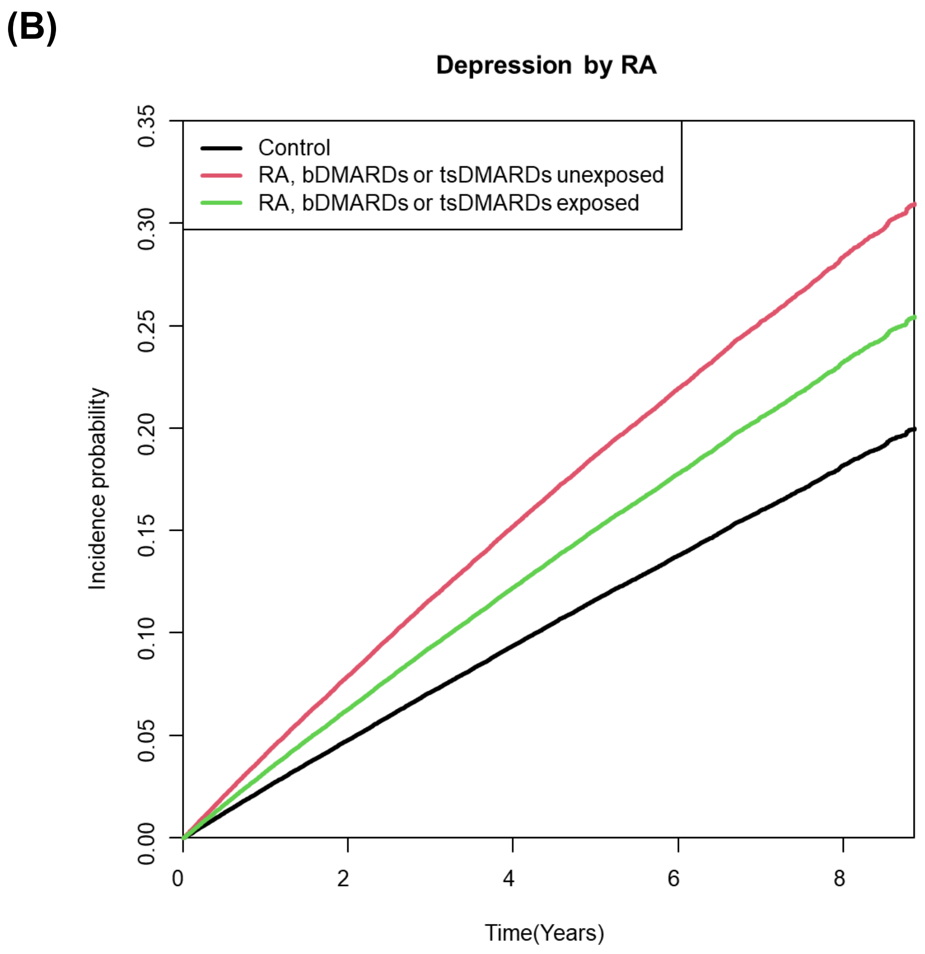

© 2024 Jeon KH et al. *JAMA Network Open.*
