# Supplementary material for: Rheumatoid Arthritis and Risk of Depression in South Korea
Source: JAMA Netw Open. 2024 Mar 5;7(3):e241139. doi: 10.1001/jamanetworkopen.2024.1139 (PMC10915683; doi:10.1001/jamanetworkopen.2024.1139)
Supplement: Supplement 2. — Data Sharing Statement [file jamanetwopen-e241139-s002.pdf]

Data Sharing Statement

Jeon. Rheumatoid Arthritis and Risk of Depression in South Korea. *JAMA Netw Open*. Published March 05, 2024. doi:10.1001/jamanetworkopen.2024.1139

Data
Data available: No
